# Impact of transit-time flow measurement on early postoperative outcomes in total arterial coronary revascularization with internal thoracic arteries: a propensity score analysis on 910 patients

**DOI:** 10.1093/icvts/ivac065

**Published:** 2022-03-03

**Authors:** Mojgan Laali, Nathalie Nardone, Pierre Demondion, Cosimo D'Alessandro, Paul Guedeney, Eleodoro Barreda, Guillaume Lebreton, Pascal Leprince

**Affiliations:** 1 Thoracic and Cardiovascular Surgery Department, Sorbonne Université, APHP, Groupe hospitalier Pitié-Salpétrière, Institute of Cardiology, Paris, France; 2 Cardiology Department, Sorbonne Université, APHP, Groupe hospitalier Pitié-Salpétrière, Institute of Cardiology, Paris, France

**Keywords:** Transit-time flow measurement, Total arterial coronary artery bypass grafting, Internal thoracic arteries, Propensity score analysis

## Abstract

**OBJECTIVES:**

The aim of this study was to evaluate the impact of transit-time flow measurement (TTFM) on early postoperative outcomes in total arterial coronary revascularization.

**METHODS:**

A single-centre retrospective analysis was conducted on 910 patients undergoing isolated total arterial coronary artery bypass grafting with internal thoracic arteries (ITAs) at our institution, between January 2017 and February 2020. Complete arterial revascularization with bilateral ITAs with a Y-configuration, or single ITA, was planned for all patients. According to the surgeon preference, TTFM was assessed in 430 patients (TTFM group). They were compared with 480 patients without TTFM assessment (no TTFM group). Primary end point was the occurrence of in-hospital major cardiac adverse events (MACE). A propensity score analysis with an inverse probability weighting approach was performed to control for selection bias.

**RESULTS:**

TTFM was associated with longer cardiopulmonary bypass times (76.0 [62.0; 91.2] vs 79.0 [65.0; 94.0] min, *P* = 0.042). Six (1.4%) patients in the TTFM group versus no patient in the no TTFM group underwent intraoperative graft revision because of unsatisfying flow values (*P* = 0.011). MACE were significantly lower in the TTFM group (14, 3.3%) than in the no TTFM group (33, 6.9%, *P* = 0.014). At crude regression, TTFM was protective against MACE occurrence (odds ratios 0.46, 95% confidence interval 0.23–0.85, *P* = 0.016). Inverse probability weighting adjustment did not significantly displace *P*-values and odds ratios for MACE occurrence in the TTFM group 0.44, 95% confidence interval 0.28–0.69, *P* < 0.001.

**CONCLUSIONS:**

Even if associated with longer cardiopulmonary bypass times, intraoperative graft flow measurement with TTFM reduces MACE occurrence and it should be recommended for graft evaluation in arterial coronary artery bypass grafting surgery.

**Figure:**
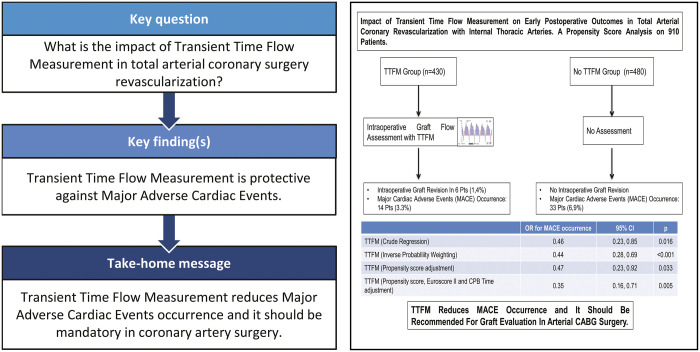

## INTRODUCTION

Since the first coronary artery bypass grafting (CABG) surgery over 5 decades ago, reducing perioperative adverse events and improving graft patency have obsessed surgeons.

Transit-time flow measurement (TTFM) [1] is a recently revived technology that allows easy assessment of graft flow (GF) and immediate revision of the graft where the results are not optimal. According to the 2018 recommendations of the European Society of Cardiology/and the European Society of Cardiothoracic Surgery, Doppler control of coronary bypass surgery is recommended. However, this recommendation does not obtain more than IIa-B rank for the lack of solid scientific evidence [2].

Published data on these techniques are generally limited to small number of cases and not comparative; therefore, their applicability to a larger cohort of patients is not immediately apparent.

Furthermore, data are still controversial: while some authors have shown a positive effect of TTFM on early and mid-term results after coronary revascularization [3, 4], other studies [5, 6] failed to demonstrate any impact of this measurement on CABG outcomes. To overcome this limitation, we set up a comparative retrospective study of patients undergoing primary isolated CABG to determine the clinical impact of TTFM on early postoperative outcomes, by the ability of TTFM to detect technical errors in coronary artery anastomoses, and the incidence of this problem.

## PATIENTS AND METHODS

### Ethic statement

An Institutional Review Board grant was released by the ethical committee of the French Society of Thoracic and Cardiovascular Surgery (EPICARD): DELIBERE_CERC-SFCTCV-2021-12_num32_LAALI arterial coronary revascularization.

### Study population

A single-centre retrospective analysis involved 945 patients undergoing isolated total arterial CABG with internal thoracic arteries (ITAs) at our institution, between January 2017 and February 2020. Preoperative patient’s data and outcomes were prospectively collected in the EPICARD database. EPICARD database was accessed for patient data extraction in March 2020. Thirty-five patients, with high-risk preoperative status according to the EuroSCORE II definition or redo procedures, were excluded from the analysis.

On-pump complete arterial revascularization with bilateral ITAs with a Y-configuration, or single ITA in case of isolated left anterior descending artery disease, was planned for all patients. All ITAs were skeletonized. Anastomosis was done with 8–0 polypropylene (Prolene; Ethicon, Somerville, NJ, USA) sutures.

Depending on the surgeon's preference, TTFMs were evaluated in 430 patients (TTFM group), or not evaluated in 480 patients (no TTFM group). An institutional prospective digital database system (DxCare, Medasys Corporation) was also used as complementary data source.

### Definitions and outcomes

In this study, the primary end point was the occurrence of in-hospital major cardiac adverse events (MACE), including in-hospital mortality, perioperative myocardial infarction, cardiac arrest, need for intra-aortic balloon pump or extra-corporeal membrane oxygenation support and the need for urgent postoperative coronary angiogram and postoperative percutaneous or surgical revascularization. The diagnosis of perioperative myocardial infarction was made on the basis of clinical symptoms and additional cardiac biomarkers with or without abnormal EKG. No follow-up was available for outcomes analysis.

All clinical events were recorded.

### Transit-time flow measurement assessment

In the TTFM group, the measurements was performed with the MiraQ or VeriQ C devices (Medistim ASA, Surgitech, France), after cross-clamp release, on partial cardiopulmonary bypass (CPB) (Video 1).

The systolic blood pressure at the time of the measurements was at least 100 mmHg.

Measurements were done in the middle portions for ITA grafts. The measurements were taken after all grafts were completed; the 2- or 3-mm probe was most commonly used. We do the measurements in 2 steps for sequential anastomosis: in the first step, we put the probe before sequential anastomosis while putting a vascular clamp after sequential anastomosis; by this way, we control the quality of anastomosis. In the second step, to verify the competitive flow, we do the measurement without clamping the distal anastomosis.

**Figure 1:**
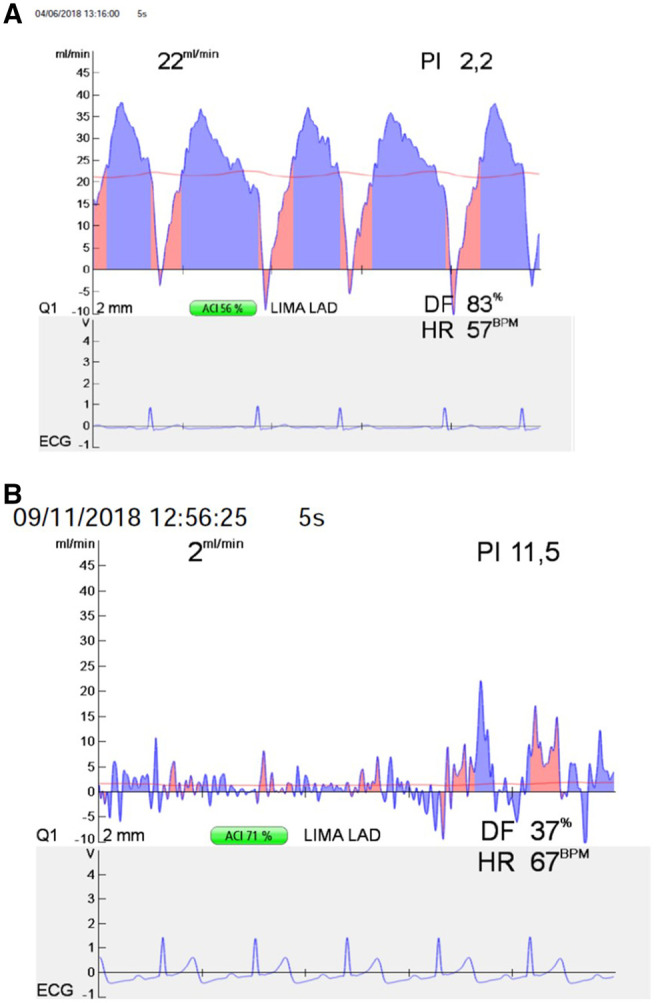
(**A**) Transit-time flow measurement assessment of a left internal thoracic artery to left anterior descending artery graft, showing good pulsatility index, graft flow and diastolic flow values. (**B**) Transit-time flow measurement assessment of a left internal thoracic artery to left anterior descending artery graft, showing unsatisfying pulsatility index, graft flow and diastolic flow values. Anastomosis will be revised.

Manipulation of grafts during measurement must be very careful to avoid any inadvertent traction on the grafts and anastomotic tears. To standardize [7] and optimize the evaluation of the TTFM, we collected the measurements that were carried out respecting the following instructions, the acoustic coupling index must be above 40% (green or yellow), indicating the accuracy of the ultrasonic conductivity; and the flow measurement was collected when mean flow indicated by the red line was constant and horizontal (Fig. 1a).

**Video 1:** Perioperative transit-time flow measurement assessment.

The patency of the grafts was assessed using 3 variables: diastolic flow (DF) curve, mean flow and pulsatility index (PI). Normally, the flow curve will show a small backflow during early systole and a predominantly forward flow during diastole [1].

Mean flow should be cautiously interpreted, as its value is not necessarily a good indicator of the quality of the anastomosis. Mean flow is largely dependent on the quality of the native coronary artery, and low mean flow can be expected in fully patent anastomoses whenever the target territory has a poor runoff. We considered mean flows of <15 ml/min to be questionable [8].

The PI is a good indicator of the flow pattern and, consequently, of the quality of the anastomosis. This number is obtained by dividing the difference between the maximum and minimum flows by the value of the mean flow. The PI value should ideally be between 1 and 5. The possibility of a technical error in the anastomosis increases for higher PI values [9].

By definitions surgical revision means revision of the distal anastomosis or revision of the graft conduit due to abnormal TTFM results (Fig. 1b).

Threshold values of TTFM assessment were mean GF >15 ml/min, PI <5 and DF >70% for left coronary bed and >50% for the right one [7].

### Statistical analyses

Comparison between the TTFM and non-TTFM groups and univariable analysis for the assessment of potential prognostic factors of MACE occurrence were performed using Chi^2^ and Fisher’s exact tests for categorical variables and Welch *t*-test for quantitative ones. A propensity analysis was performed to control for selection bias. An inverse probability weighting (IPW) approach was performed to reduce potential differences between the TTFM and non-TTFM sub-cohorts in baseline characteristics. The propensity score was obtained with a multivariable non-parsimonious logistic regression model estimating, for each patient, the probability to receive a TTFM assessment using the preoperative features. The preoperative features used to model the propensity score were: sex, age, body mass index, creatinin clearance (ml/min), chronic dialysis, diabetes, chronic obstructive pulmonary disease, cerebrovascular arteriopathy, neuromuscular impairment, peripheral artery disease, previous or planned abdominal aortic operation, extracardiac arteriopathy, NYHA class, CCS IV angina, recent MI (<90 days), LVEF (%), pulmonary artery pressure, urgency status, left main coronary artery stenosis >50%, previous percutaneous transluminal coronary angioplasty, hypertension, dyslipidaemia, vitamin K antagonists treatment, platelet antiaggregant treatment, double platelet antiaggregant treatment and number of arterial conduits. All variables included in the propensity score model reflected knowledge available at baseline. Standardized differences were examined to assess balance provided by the obtained propensity score weighting, with a threshold of 10% designated to indicate clinically meaningful imbalance (forestplot). In the primary analysis, the propensity score was used in the modelling of MACE occurrence by TTFM assessment through an IPW approach. A first sensitivity analysis was conducted to assess the robustness of the findings using the propensity score as an adjustment variable in a multivariable regression explaining MACE by TTFM. In a second set of sensitivity analyses, variables significantly associated to MACE in univariable analyses were additionally added to the adjustment model: CPB time (a clinically relevant risk factor) and EuroSCORE II (a composite score containing the other significant variables).

Data are expressed as median [Q1; Q3] for continuous variables, and percentages for qualitative variables. All *P*-values were two-tailed.

Descriptive statistical analyses were carried out on the site https://www.pvalue.io (Medistica., pvalue.io, a graphic user interface to the R statistical analysis software for scientific medical publications., 2020). R software 4.0.3 was used for univariable and multivariable logistic regressions.

### Data availability statement

The data underlying this article will be shared on reasonable request to the corresponding author.

## RESULTS

Preoperative characteristics are summarized in Table 1. Operative data and postoperative outcomes were collected as found in Table 2. The CPB times were longer in the TTFM group because of the supplementary time needed for measurement (76.0 min [62.0; 91.2] vs 79.0 min [65.0; 94.0], *P* = 0.042).

**Table 1:** Preoperative characteristics

	No TTFM (*n* = 480)	TTFM (*n* = 430)	*P*-Value
Age, median [Q1; Q3]	66 [60.0; 73.0]	66 [60.0; 72.0]	0.526
Sex, female, *n* (%)	74 (15)	62 (14)	0.673
Body mass index, median [Q1; Q3]	26.6 [24.2; 30.1]	26.9 [24.1; 29.8]	0.627
Hypertension, *n* (%)	349 (73)	299 (70)	0.291
Diabetes, *n* (%)	190 (40)	188 (44)	0.206
Dyslipidaemia, *n* (%)	369 (77)	310 (72)	0.098
Insulin-dependent diabetes, *n* (%)	52 (11)	58 (13)	0.220
Creatinin clearance (ml/min), median [Q1; Q3]	87.0 [65.0; 110]	74.8 [42.0; 98.9]	<0.001
Chronic dialysis, *n* (%)	13 (2.7)	7 (1.6)	0.267
LVEF%, median [Q1; Q3]	55.0 [48.0; 60.0]	57.5 [50.0; 61.0]	0.148
LVEF% ≤20, *n* (%)	3 (0.62)	6 (1.4)	0.321
LVEF% 21–30, *n* (%)	21 (4.4)	14 (3.3)	0.381
LVEF% 31–50, *n* (%)	154 (32)	114 (27)	0.066
Pulmonary artery pressure, median [Q1; Q3]	28.0 [20.0; 30.0]	28.0 [20.0; 29.0]	0.817
Previous PTCA, *n* (%)	163 (34)	93 (22)	<0.001
LMCA stenosis >50%, *n* (%)	227 (47)	181 (42)	0.115
Peripheral artery disease, *n* (%)	79 (16)	48 (11)	0.021
Cerebrovascular arteriopathy, *n* (%)	46 (9.6)	14 (3.3)	<0.001
Previous/planned abdominal aortic operation, *n* (%)	6 (1.2)	5 (1.2)	0.904
Extracardiac arteriopathy, *n* (%)	110 (23)	51 (12)	<0.001
COPD, *n* (%)	25 (5.2)	22 (5.1)	0.950
Neuromuscular impairment, *n* (%)	55 (11)	19 (4.4)	<0.001
Urgent status, *n* (%)			0.091
Elective	286 (60)	280 (65)	
Urgent	154 (32)	114 (27)	–
Emergency	29 (6)	15 (3.5)	–
CCS IV angina, *n* (%)	92 (19)	65 (15)	0.106
Recent MI (<90 days), *n* (%)	95 (20)	65 (15)	0.064
NYHA class, *n* (%)			
I	193 (40)	241 (56)	<0.001
II	235 (49)	152 (35)	–
III	48 (10)	31(7.2)	–
IV	4 (0.83)	6 (1.4)	–
Number of diseased vessels, *n* (%)			
1	20 (4.17)	11 (2.6)	0.355
2/	70 (15)	69 (16)	–
3	374 (78)	343(80)	–
Unknown	16 (3.3)	7 (1.6)	
Platelet antiaggregant therapy, *n* (%)	425 (89)	391 (91)	0.237
Double platelet antiaggregant therapy, *n* (%)	128 (27)	106 (25)	0.487
Vitamin K antagonist therapy, *n* (%)	31 (6.5)	21 (4.9)	0.307
EuroSCORE II, median [Q1; Q3]	1.43 [0.915; 2.34]	1.44 [0.881; 2.32]	0.650

LVEF: left ventricular ejection fraction; COPD: chronic obstructive pulmonary disease; LMCA: left main coronary artery; PTCA: percutaneous transluminal coronary angioplasty; TTFM: transit-time flow measurement; NYHA: New-York Heart Association; CCS: Canadian Cardiovascular Angina Grade; MI: myocardial infarction.

**Table 2:** Operative data and postoperative outcomes

	No TTFM (*n* = 480)	TTFM (*n* = 430)	*P*-Value
LITA, *n* (%)	56 (12)	35 (8.1)	0.077
BITA, *n* (%)	424 (88)	395 (92)	–
Number of anastomoses/patient, median [Q1; Q3]	4.00 [3.00; 4.00]	4.00 [3.00; 4.00]	0.795
CPB time (min), median [Q1; Q3]	76.0 [62.0; 91.2]	79.0 [65.0; 94.0]	0.042
Cross-clamp time (min), median [Q1; Q3]	63.0 [50.0; 78.0]	63.0 [50.0; 78.0]	0.784
Intraoperative graft revision because unsatisfying TTFM values, *n* (%)	0 (0)	6 (1.4)	0.011
IABP, *n* (%)	9 (1.9)	6 (1.4)	0.570
ECMO, *n* (%)	12 (2.5)	6 (1.4)	0.232
Postoperative MI, *n* (%)	7 (1.5)	4 (0.9)	0.467
Cardiac arrest, *n* (%)	11 (2.3)	3 (0.7)	0.051
Urgent coronary angiogram, *n* (%)	12(2.5)	7 (1.6)	0.358
Postoperative revascularization, *n* (%)	8 (1.7)	4 (0.9)	0.394
PTCA, *n* (%)	7 (1.5)	2 (0.47)	0.183
Surgical, *n* (%)	1 (0.21)	2 (0.47)	0.605
In-hospital mortality, *n* (%)	15 (3.1)	7 (1.6)	0.142
MACE	33 (6.9)	14 (3.3)	0.014
Cardiac-related mortality	9 (1.9)	3 (0.7)	0.120

CPB: cardiopulmonarybypass;ECMO: extra-corporeal membrane oxygenation; IABP: intra-aortic balloon pump; LITA: left internal thoracic artery; BITA: bilateral internal thoracic arteries; MACE: major cardiac adverse events; PTCA: percutaneous transluminal coronary angioplasty; TTFM: transit-time flow measurement.

Six (1.4%) patients in the TTFM group vs no patients in the No TTFM group underwent intraoperative graft revision because of unsatisfying flow values, *P* = 0.011: left ITA to left anterior descending artery in 3 patients, right ITA to posterior descending artery in 2 patients and right ITA to obtuse marginal artery in 1 patient. No electrocardiographic changes were noted in those patients in whom unsatisfactory GF was noticed and the anastomosis had to be corrected. In all cases, the anastomotic problem were corrected at the time of surgery, resulting in satisfactory flows. There were no kinked or twisted conduits. Postoperative course of all these patients was uneventful. The mean GF, PI and DF values of these patients before and after revision were not available for analysis.

MACE were significantly lower in the TTFM group (14, 3.3%) than in the no TTFM group (33, 6.9%, *P* = 0.014). Twelve (2.5%) patients in the no TTFM group vs 7 (1.6%, *P* = 0.36) in the TTFM group underwent unplanned postoperative coronary angiogram. Details of coronary angiograms results are resumed in Table 3. TTFM values were available for 4/6 patients with abnormal coronary angiograms: mean GF, PI, and DF were 33 ml/min, 3, and 69%. Eight patients in the no TTFM group and 4 patients in the TTFM group underwent urgent immediate postoperative revascularization; details of revascularization modalities were summarized in the same table. The remaining patients (3 patients in the no TTFM group and 2 patients in the TTFM group) were medically treated.

**Table 3:** Details of postoperative coronary angiograms

	No TTFM	TTFM
Coronary angiograms	12	7
Indication ECG/troponine elevation	5	2
Cardiogenic shock	7	5
Normal angiogram	1	1
Native vessels stenosis/occlusion	1	1
Graft stenosis/occlusion	10	5

TTFM: transit-time flow measurement.

Cardiac-related mortality was 1.9% (9 patients) in the no TTFM group vs 0.7% (3 patients, *P* = 0.12) in the TTFM group. Non-cardiac causes of death in the no TTFM group were septic shock (3), hypoxic arrest (2) and cerebro-vascular accident (1). Non-cardiac causes of death in the TTFM group were cerebro-vascular accident (3) and mesenteric ischaemia in 1 patient.

Details of construction of propensity score and univariable and multivariable regressions are available as Supplementary Material.

At crude regression, TTFM was protective against MACE occurrence (odds ratios 0.46, 95% confidence interval 0.23–0.85, *P* = 0.016). IPW adjustment did not significantly displace *P-*values and odds ratios for MACE occurrence in the TTFM group 0.44, 95% confidence interval 0.28–0.69, *P* < 0.001. Multivariable regressions are resumed in Table 4.

**Table 4:** Multivariable regressions for major cardiac adverse event occurrence

	Odds ratio [95% CI]	*P*-Value
Crude regression		
TTFM = 1	0.46 [0.23; 0.85]	0.016
IPW regression		
TTFM = 1	0.44 [0.28; 0.69]	<0.001
Regression for MACE adjusted on propensity score		
TTFM = 1	0.47 [0.23; 0.92]	0.033
Propensity score	0.78 [0.14; 4.18]	0.772
Regression for MACE adjusted on propensity score, EuroSCORE II and CPB time		
TTFM = 1	0.35 [0.16; 0.71]	0.005
EuroSCORE II	1.29 [1.18; 1.40]	<0.001
CPB time (min)	1.01 [1.00; 1.02]	0.097
Propensity score	3.50 [0.58; 21.3]	0.172

CI: confidence interval; CPB: cardiopulmonary bypass; MACE: major cardiac adverse events; TTFM: transit-time flow measurement.

## DISCUSSION

TTFM gives important and accurate intraoperative information about the status and patency of each individual graft. It enables technical problems such as kinked, twisted or stenotic grafts to be diagnosed accurately, thereby allowing prompt revision of the constructed grafts before the patient leaves the operating room [10]. Thus, haemodynamic instability during the early postoperative period, which could be catastrophic, is possibly prevented, and the probability of early graft failure is minimized significantly, improving the outcome of CABG surgery.

However, its usefulness during a CABG is not yet unanimous; in one hand, some surgeons argue against the use of TTFM, saying that TTFM is difficult to use and is time-consuming. That might be right in the beginning, but the learning curve is very short and requires no more than 30 s per measurement [11].

In addition, some believe that TTFM is unnecessary, arguing that incidences of surgical mistakes are extremely low, and the high cost of the equipment causing surgeons’ and hospitals’ hesitation in adopting TTFM technology.

On the other hand, even if the threshold values and curves were defined for different types of grafts and revascularized vessels [12], standardization of TTFM findings is difficult because of large biologic variability among different patients, as well as within the same patient. Interpretation of flow curves and TTFM findings is largely dependent on the surgeon’s personal experience.

The ability to correctly interpret TTFM findings develops with clinical and experimental experience and, thus, surgeons who have not been exposed to TTFM technology cannot easily accord it the proper level of importance.

Finally, manipulation of grafts during measurement, which must be very careful in order to avoid any inadvertent traction on the grafts and anastomotic tears, is another reason that restraint the surgeons.

As stated in the Patients and Methods section, in the TTFM group, the measurements were performed after cross-clamp release, on partial CPB.

Since coronary bypass strategy in our centre is all-arterial with 2 ITAs, we changed our strategy of TTFM measurement: instead of testing on-pump after clamp release, now we do the measurement for each anastomosis when we are still under clamping, to facilitate both technical factors and difficulties for interpretation of the data. Measurements during ‘cross-clamp on’ can help us easily check the quality of anastomosis specially based on the PI value. This new practice has made most surgeons more confident.

Of course, the measurements should be done again after cross-clamp release, and even more after weaning the CPB, to ensure that the lengths of the grafts and the geometry of the sequential anastomosis are correct, and to give us information about competitive flow [13]. Nevertheless, sometimes it could be very difficult to expose anastomoses on lateral or inferior wall after coming off pump, especially with multiple sequential arterial grafts. In such cases, we rely upon TTFM only assessed while on partial CPB.

Occurrence of MACE in contemporary coronary surgery is rare, ranging between 2% and 7% postoperatively [5, 12] and 12% at 5 years [14]. In our series, the overall occurrence of MACE was 5%.

In comparative studies, Becit *et al.* [4] and Bauer *et al.* [15] reported significant improvement of short-term clinical operative outcomes using TTFM. Recently, a large meta-analysis [10] showed that TTFM improves CABG procedures; this evidence was supported by the REQUEST study [12], a large prospective multicentre study that investigated surgical procedure changes guided by TTFM and ultrasonic imaging of aorta, conduits and grafts. On the other hand, the GRIIP RCT trial [5] and a recent sub-analysis of ROOBY trial [6] failed to demonstrate any impact of TTFM on 1 and 5-years clinical outcomes. Actually, graft assessment in the GRIIP trial involved TTFM in association with intraoperative angiography, resulting in a more difficult interpretation of the unique effect of TTFM on postoperative results. In the ROOBY trial sub-analysis, TTFM was associated with a significant improvement of graft patency (83% vs 78%, *P* < 0.01) and fewer occluded grafts (29%, vs 38%, *P* = 0.01).

In our series, thanks to adoption of TTFM, MACE occurrence was significantly reduced by half, dropping from 6.9% to 3.3%. Moreover, every adverse event was reduced, even without reaching statistical significance. Our hypothesis is that intraoperative graft revision in the TTTFM group may have positively affected the outcomes; a technical issue on conduit or graft anastomosis may be more dramatic in multiple sequential arterial coronary surgery, since blood perfusion of a large amount of myocardial wall often relies upon the flow in a single conduit.

Adverse cardiac events in TTFM patients could be explained because even if the threshold values and curves were defined for different types of grafts and revascularized vessels, standardization of TTFM findings is difficult because of large biologic variability among different patients, as well as within the same patient. Moreover, delayed occlusion of grafts or anastomosis, for example because of left ITA intimal tears leading in later thrombosis. As advocated by Di Giammarco *et al.* [16], addition of high-resolution epicardial ultrasonography could increase the accuracy of TTFM assessment.

The ability to correctly interpret TTFM findings develops with clinical and experimental experience, and we believe that, even with these limitations, by using more and more this device, we can prevent a large number of unpleasant events. On the other hand, we have to keep in mind that TTFM values are only useful and do not dictate the decision. Even if TTFM values could suggest the revision of the anastomosis, surgeon's experience will always have the last word.

### Limitations

There are some limitations in this present study. As stated in the methods section, this study carries all the limits that a retrospective design implies, even if all data were prospectively collected into the database of EPICARD. Unfortunately, mean GF, PI and DF values were not available for the whole study population, as well as for patients undergoing anastomosis revision because of unsatisfying TTFM values. When we talk about the surgeon's preference, it means that when our centre was equipped with this device in 2015, half of the surgeons was just not convinced of the usefulness of this device, and they never used that. Even if we think that there is not any indication bias in this study, we agree that a bias by indication cannot be ruled out, also because of the non-randomized design of the study. Now, after presenting the results in our centre, all the surgeons adopted the TTFM for graft evaluation and randomized study is no longer possible in our centre. Comparison between the no TTFM and TTFM patients included a propensity score analysis, to control for selection bias. Once the propensity model is constructed and a propensity score is calculated for each patient, 3 common types of comparison are employed: matching, stratification and multivariable adjustment [17]. Among common types of comparison suggested [17], we used the propensity score as multivariable adjustment with a IPW approach [18, 19]; this was the sole mean of adjusting because of the small number of events, since no patients needed to be excluded from the analysis.

Further randomized studies have been advocated [6], but, in our opinion, nowadays, randomization is no more ethically possible and even desirable. First, quality control assessment of anastomoses is recommended by recent ESC guidelines [2]. Furthermore, surgical teams confident with TTFM have a daily proof of usefulness of the technique and its advantages for all coronary patients. Such surgeons, just like us, will be very reluctant to abandon a tool that allows reduction of adverse events in their patients.

Therefore, we believe that as the push towards better patient care becomes increasingly important, the use of TTFM during CABG will become imperative. So, large comparative studies like ours could be very helpful.

## CONCLUSION

In this series, intraoperative GF measurement with TTFM was associated with a reduction of MACE occurrence. According to our experience, 3 stages of TTFM measurement provide valuable confidence in anastomotic patency during CABG, and it should be recommended for graft evaluation in arterial CABG surgery.

## SUPPLEMENTARY MATERIAL

Supplementary material is available at *ICVTS* online.

## Supplementary Material

ivac065_Supplementary_Data

## Notes

Presented at the 35th Annual Meeting of the European Association for Cardio-Thoracic Surgery, Barcelona, Spain, 13–16 October 2021.

## ABBREVIATIONS

CABG: Coronary artery bypass grafting
CPB: Cardiopulmonary bypass
DF: Diastolic flow
EPICARD: French Society of Thoracic and Cardiovascular Surgery
GF: Graft flow
IPW: Inverse probability weighting
ITAs: Internal thoracic arteries
MACE: Major cardiac adverse events
PI: Pulsatility index
TTFM: Transit-time flow measurement
