# Supplementary material for: Impact of transit-time flow measurement on early postoperative outcomes in total arterial coronary revascularization with internal thoracic arteries: a propensity score analysis on 910 patients
Source: Interact Cardiovasc Thorac Surg. 2022 Mar 3;35(2):ivac065. doi: 10.1093/icvts/ivac065 (PMC12083446; doi:10.1093/icvts/ivac065)
Supplement: ivac065_Supplementary_Data [file ivac065_supplementary_data.docx]

Table 1: Univariate regressions for TTFM before IPW

| Characteristic | N | OR^1^ | 95% CI^1^ | p-value |
| --- | --- | --- | --- | --- |
| **Sex** | 910 | 1.08 | 0.75, 1.56 | 0.673 |
| **Age** | 910 | 1.00 | 0.98, 1.01 | 0.526 |
| **BMI** | 910 | 0.99 | 0.97, 1.02 | 0.627 |
| **Creatinin Clearance (mL/min)** | 910 | 0.99 | 0.98, 0.99 | **<0.001** |
| **Chronic dialysis** | 910 | 0.59 | 0.22, 1.47 | 0.272 |
| **Diabetes** | 910 | 1.19 | 0.91, 1.54 | 0.206 |
| **COPD** | 910 | 0.98 | 0.54, 1.77 | 0.950 |
| **Cerebrovascular Artheriopathy** | 910 | 0.32 | 0.17, 0.57 | **<0.001** |
| **Neuromuscular impairment** | 910 | 0.36 | 0.20, 0.60 | **<0.001** |
| **Peripheral artery disease** | 910 | 0.64 | 0.43, 0.93 | **0.022** |
| **Previous or planned abdominal aortic operation** | 910 | 0.93 | 0.27, 3.11 | 0.904 |
| **Extracardiac artheriopathy** | 910 | 0.45 | 0.31, 0.65 | **<0.001** |
| **NYHA** | 910 | 0.68 | 0.56, 0.83 | **<0.001** |
| **CCS IV Angina** | 910 | 0.75 | 0.53, 1.06 | **0.107** |
| **Recent MI (<90 days)** | 910 | 0.72 | 0.51, 1.02 | **0.065** |
| **LVEF (%)** | 910 | 1.01 | 1.00, 1.02 | **0.148** |
| **Pulmonary artery pressure** | 910 | 1.00 | 0.98, 1.02 | 0.817 |
| **Urgency status** | 910 | 0.79 | 0.63, 0.99 | **0.038** |
| **LMCA stenosis >50%** | 910 | 0.81 | 0.62, 1.05 | **0.116** |
| **Previous PTCA** | 910 | 0.54 | 0.40, 0.72 | **<0.001** |
| **Hypertension** | 910 | 0.86 | 0.64, 1.14 | 0.291 |
| **Dyslipidemia** | 910 | 0.78 | 0.58, 1.05 | **0.098** |
| **Vitamin K – Antagonist Treatment** | 910 | 0.74 | 0.42, 1.31 | 0.308 |
| **Antiaggregant Treatment** | 910 | 1.30 | 0.84, 2.01 | 0.238 |
| **Double Antiaggregant Treatment** | 910 | 0.90 | 0.67, 1.21 | 0.487 |
| **N arterial conduits** | 910 | 1.49 | 0.96, 2.34 | **0.078** |
| ^1^OR = Odds Ratio, CI = Confidence Interval | | | | |

Table 2: Exposure model (propensity score) - Multivariate regression

| Characteristic | OR^1^ | 95% CI^1^ | p-value |
| --- | --- | --- | --- |
| **Sex** | 1.27 | 0.85, 1.90 | 0.244 |
| **Age** | 0.97 | 0.95, 0.99 | **<0.001** |
| **BMI** | 1.03 | 1.00, 1.07 | 0.059 |
| **Creatinin Clearance (mL/min)** | 0.98 | 0.98, 0.99 | **<0.001** |
| **Chronic dialysis** | 0.26 | 0.08, 0.72 | **0.012** |
| **Diabetes** | 1.42 | 1.05, 1.94 | **0.024** |
| **COPD** | 1.13 | 0.58, 2.20 | 0.721 |
| **Cerebrovascular Artheriopathy** | 1.66 | 0.41, 7.23 | 0.486 |
| **Neuromuscular impairment** | 0.41 | 0.12, 1.28 | 0.135 |
| **Peripheral artery disease** | 1.63 | 0.73, 3.74 | 0.240 |
| **Previous or planned abdominal aortic operation** | 1.39 | 0.34, 5.50 | 0.638 |
| **Extracardiac artheriopathy** | 0.36 | 0.15, 0.85 | **0.021** |
| **NYHA** | 0.65 | 0.51, 0.82 | **<0.001** |
| **CCS IV Angina** | 1.00 | 0.65, 1.54 | 0.990 |
| **Recent MI (<90 days)** | 0.75 | 0.48, 1.16 | 0.200 |
| **LVEF (%)** | 1.00 | 0.98, 1.01 | 0.610 |
| **Pulmonary artery pressure** | 1.00 | 0.98, 1.03 | 0.917 |
| **Urgency status** | 0.94 | 0.69, 1.28 | 0.703 |
| **LMCA stenosis >50%** | 1.06 | 0.79, 1.43 | 0.684 |
| **Previous PTCA** | 0.52 | 0.37, 0.73 | **<0.001** |
| **Hypertension** | 0.81 | 0.57, 1.14 | 0.221 |
| **Dyslipidemia** | 0.86 | 0.60, 1.23 | 0.413 |
| **Vitamin K – Antagonist Treatment** | 0.77 | 0.40, 1.44 | 0.416 |
| **Antiaggregant Treatment** | 1.77 | 1.08, 2.94 | **0.025** |
| **Double Antiaggregant Treatment** | 1.11 | 0.77, 1.61 | 0.574 |
| **N arterial conduits** | 1.51 | 0.93, 2.50 | 0.101 |
| ^1^OR = Odds Ratio, CI = Confidence Interval | | | |

Area Under the Curve = 0.7140795

**
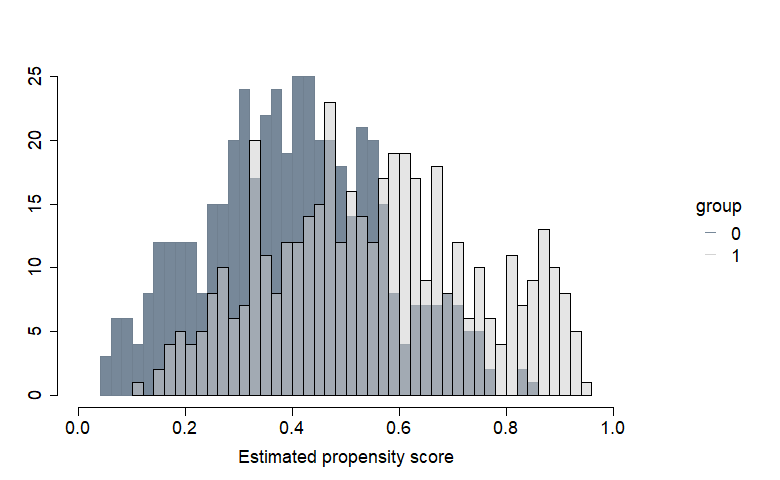
**

*Graph 1: Distribution of the estimated propensity score in the TTFM (1) and non-TTFM (2) groups*

- 1. **Inverse Probability Weighting (IPW) Adjustment**


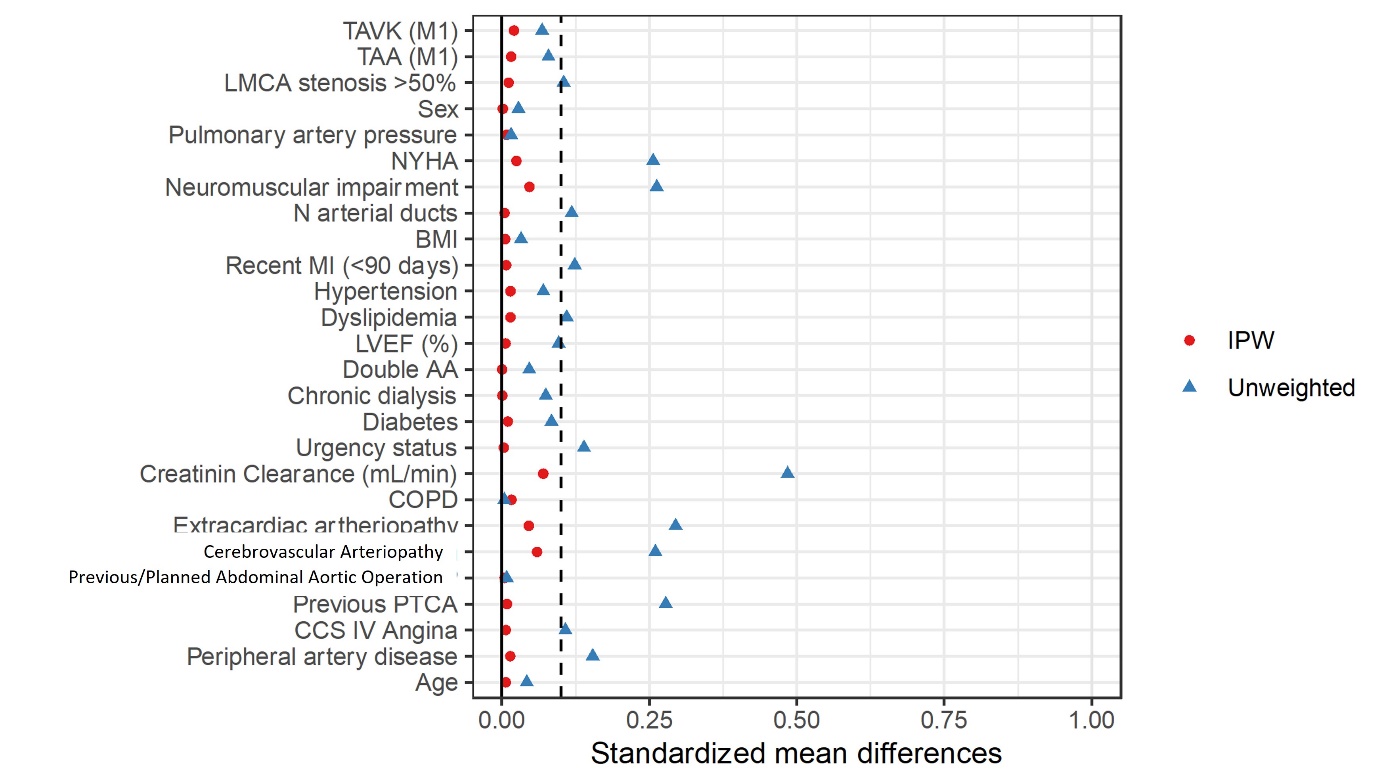


*Graph 2: Standardized mean differences in the weighted and unweighted sample*

Table 3: Univariate regressions for MACE

| Characteristic | N | OR^1^ | 95% CI^1^ | p-value |
| --- | --- | --- | --- | --- |
| **TTFM** | 910 | 0.46 | 0.23, 0.85 | **0.016** |
| **Sex** | 910 | 0.55 | 0.28, 1.17 | 0.099 |
| **Age** | 910 | 1.03 | 1.00, 1.07 | **0.044** |
| **BMI** | 910 | 0.98 | 0.92, 1.04 | 0.529 |
| **Creatinin Clearance (mL/min)** | 910 | 0.99 | 0.99, 1.00 | **0.033** |
| **Chronic dialysis** | 910 | 6.73 | 2.11, 18.3 | **<0.001** |
| **Diabetes** | 910 | 1.14 | 0.63, 2.06 | 0.654 |
| **COPD** | 910 | 0.81 | 0.13, 2.73 | 0.773 |
| **Cerebrovascular Artheriopathy** | 910 | 0.62 | 0.10, 2.07 | 0.511 |
| **Neuromuscular impairment** | 910 | 0.49 | 0.08, 1.63 | 0.328 |
| **Peripheral artery disease** | 910 | 1.72 | 0.79, 3.43 | 0.141 |
| **Previous or planned abdominal aortic operation** | 910 | 1.85 | 0.10, 9.99 | 0.560 |
| **Extracardiac artheriopathy** | 910 | 1.11 | 0.49, 2.24 | 0.788 |
| **NYHA** | 910 | 2.26 | 1.55, 3.29 | **<0.001** |
| **CCS IV Angina** | 910 | 2.64 | 1.38, 4.90 | **0.002** |
| **Recent MI (<90 days)** | 910 | 2.58 | 1.34, 4.77 | **0.003** |
| **LVEF (%)** | 910 | 0.93 | 0.91, 0.95 | **<0.001** |
| **Pulmonary artery pressure** | 910 | 1.07 | 1.03, 1.11 | **<0.001** |
| **Urgency status** | 910 | 1.87 | 1.19, 2.90 | **0.006** |
| **LMCA stenosis >50%** | 910 | 1.71 | 0.95, 3.13 | 0.077 |
| **Previous PTCA** | 910 | 1.21 | 0.63, 2.24 | 0.554 |
| **Hypertension** | 910 | 1.34 | 0.69, 2.80 | 0.404 |
| **Dyslipidemia** | 910 | 0.58 | 0.32, 1.10 | 0.084 |
| **Vitamin K – Antagonist Treatment** | 910 | 2.07 | 0.69, 5.03 | 0.143 |
| **Antiaggregant Treatment** | 910 | 0.97 | 0.41, 2.85 | 0.943 |
| **Double Antiaggregant Treatment** | 910 | 1.11 | 0.56, 2.09 | 0.754 |
| **N arterial conduits** | 910 | 0.93 | 0.39, 2.74 | 0.881 |
| **Euroscore II** | 910 | 1.26 | 1.17, 1.37 | **<0.001** |
| **CPB time (min)** | 910 | 1.01 | 1.00, 1.02 | **0.028** |
| ^1^OR = Odds Ratio, CI = Confidence Interval | | | | |

Crude regression for MACE

| Characteristic | OR^1^ | 95% CI^1^ | p-value |
| --- | --- | --- | --- |
| **TTFM** |  |  |  |
| 0 | — | — |  |
| 1 | 0.46 | 0.23, 0.85 | **0.016** |
| ^1^OR = Odds Ratio, CI = Confidence Interval | | | |
|  | | | |

IPW regression for MACE

| Characteristic | OR^1^ | 95% CI^1^ | p-value |
| --- | --- | --- | --- |
| **TTFM** |  |  |  |
| 0 | — | — |  |
| 1 | 0.44 | 0.28, 0.69 | **<0.001** |
| ^1^OR = Odds Ratio, CI = Confidence Interval | | | |
|  | | | |

Sensibility analysis 1: Regression for MACE adjusted on propensity score

| Characteristic | OR^1^ | 95% CI^1^ | p-value |
| --- | --- | --- | --- |
| **TTFM** |  |  |  |
| 0 | — | — |  |
| 1 | 0.47 | 0.23, 0.92 | **0.033** |
| **Propensity score** | 0.78 | 0.14, 4.18 | 0.772 |
| ^1^OR = Odds Ratio, CI = Confidence Interval | | | |
|  | | | |
|  | | | |

Sensibility analysis 2: Regression for MACE adjusted on propensity score, Euroscore and CPB time

| Characteristic | OR^1^ | 95% CI^1^ | p-value |
| --- | --- | --- | --- |
| **TTFM** |  |  |  |
| 0 | — | — |  |
| 1 | 0.35 | 0.16, 0.71 | **0.005** |
| **Euroscore** | 1.29 | 1.18, 1.40 | **<0.001** |
| **CPB time (min)** | 1.01 | 1.00, 1.02 | 0.097 |
| **Propensity score** | 3.50 | 0.58, 21.3 | 0.172 |
| ^1^OR = Odds Ratio, CI = Confidence Interval | | | |
|  | | | |
